# Clinical implications of aberrant PD-1 expression for acute leukemia prognosis

**DOI:** 10.1186/s40001-023-01352-8

**Published:** 2023-09-27

**Authors:** Yanjie Ruan, Jiyu Wang, Qiuye Zhang, Huiping Wang, Cong Li, Xuanxuan Xu, Zhimin Zhai

**Affiliations:** 1 Department of Hematology, Hematology Research Center, The Second Hospital of Anhui Medical University, Hefei, 230601 Anhui China; 2 Department of Pathology, The Fourth Affiliated Hospital of Anhui Medical University, Hefei, 230013 Anhui China; 3 People’s Hospital of Taizhou, Fifth Affiliated Hospital of Nantong University, Taizhou, 225300 Jiangsu China; 4 Jingzhou Hospital, Tongji Medical College, Huazhong University of Science and Technology, Wuhan, 430000 Hubei China

**Keywords:** PD-1, Acute lymphoma leukemia, Acute myeloid leukemia, CD4+ T lymphocytes, CD8+ T lymphocytes, Prognosis

## Abstract

**Background:**

Acute lymphoblastic leukemia (ALL) and acute myeloid leukemia (AML) are the most common types of leukemia in adults with an overall poor prognosis. PD-1 alone or combined with other immune checkpoint blockade is a promising research direction for the treatment of acute leukemia (AL) patients. However, clinical Implications of aberrant PD-1 expression in peripheral CD4+ and CD8+ T lymphocytes of AML and ALL patients in assessing the prognosis of diseases, remains inconclusive.

**Methods:**

In the present study, we used flow cytometry to evaluate PD-1 expression on the surface of CD4+ and CD8+ T lymphocytes in the peripheral circulation of AML and ALL patients and its clinical significance. A total of 53 AML patients, 44 ALL patients and 28 healthy controls were enrolled in this study and peripheral blood specimens were detected by flow cytometry.

**Results:**

Our results indicated that percentages of CD4+ PD1+ and CD8+ PD1+ T lymphocytes in newly diagnosed and non-remission groups were significantly higher than healthy control both in AML and ALL patients. The high level of CD4+ PD1+ and CD8+ PD1+ T lymphocytes were respectively poor prognostic indicators of AML patients and ALL patients but had no significant correlation with most common clinical risks.

**Conclusions:**

Our findings show that aberrant PD-1 expression correlates with the prognosis of AL patient and may thus serve as poor prognostic indicators. Immunotherapy using PD-1 inhibitors may be a promising strategy for AML and ALL patients with peripheral circulating CD4+ PD1+ and CD8+ PD1+ T lymphocytes positively expressed, respectively.

## Introduction

Acute leukemia (AL) is a malignant clonal disease originating from hematopoietic stem cells. Abnormal blast cells and immature cells (leukemia cells) in the bone marrow proliferate in large numbers and inhibit normal hematopoiesis. It is mainly divided into acute lymphoblastic leukemia (ALL) and acute myeloid leukemia (AML) [1]. Chemotherapy is currently the main treatment option for patients with AML and ALL but is limited due to the severe side effects and drug resistance [2]. Cancer immunotherapy has recently been developed to improve the specificity and strength of the immune system against cancer. In recent years we have also witnessed a breakthrough in the field of oncology that is represented by the development of novel agents: the immune checkpoint inhibitors, which "release the brakes" of the immune system. Immune checkpoint inhibitors, which is currently a hot area of research and may have important therapeutic value [3]. Consistently, PD-1 (Programmed Cell Death Protein 1) and its ligand (PD-L1) inhibitors have been approved by FDA and have been shown to be quite effective in several neoplasms, including leukemia [4].

Almost 30 years ago, Jenkins et al. had shown that effective activation of naive T cells requires the participation of TCR (T cell receptor) and B7/CD28 signals [5]. PD-1 (Programmed death protein 1, CD279) is an inhibitor receptor that belongs to the B7/CD28 family. As early as 1992, PD-1 was identified on T cells undergoing apoptosis by Ishida Y et al. [6]. PD-1 expressed on a broad variety of cells including activated T cells, B cells, monocytes, dendritic cells, and NK cells, except for naive lymphocytes prior to activation [7, 8]. Of note, PD-1 is highly expressed on tumor-specific T cells [9]. PD-1 plays an important role in inhibiting immune responses and promoting self-tolerance through modulating the activity of T-cells, activating apoptosis of antigen-specific T cells and inhibiting apoptosis of regulatory T cells [10–12].

Recent research reported that PD-1 expression is related with poor prognosis of cancers. PD-1/PD-L1-targeted inhibitors play an important role in cancers such as breast cancer, lung cancer, colorectal cancer, gastric cancer, bladder cancer, pancreatic cancer, prostate cancer, DLBCL and so on [10, 13–15]. In this study, we evaluated the expression of PD-1 on CD4+ and CD8+ T lymphocytes in Peripheral Blood and summarized the role of PD-1 which is a crucial factor affecting the prognosis of AML and ALL patients.

## Materials and methods

### Patients

102 AL patients including 57 AML patients, 45 ALL patients and 28 healthy controls enrolled in our study were recruited from September 2016 to August 2019 in the Second Hospital of Anhui Medical University. All diagnosed patients with AML and ALL were divided into newly diagnosed (ND) group, complete remission (CR) group, and Non-remission (NR) group according to the 2016 National Comprehensive Cancer Network (NCCN) guidelines 2nd Edition. All healthy volunteers enrolled in this study have no abnormal liver and kidney function, no autoimmune diseases, no history of immunosuppressive drugs. This study was approved by the Institutional Review Board (IRB) Institutional of the Second Hospital of Anhui Medical University. All patients enrolled in the study have signed informed consent.

### PD-1 analysis

Peripheral blood mononuclear cells (PBMCs) were separated by density gradient centrifugation (Ficoll-Hypaque, Amersham Bio-sciences, Sweden), and washed with phosphate-buffered saline (PBS). After washing, 100μL PBMCs was incubated with monoclonal antibodies and analyzed by flow cytometer (CytoFLEX, Beckman Coulter, USA), and EXPO 32 Multicomp software was used for data acquisition and analysis. The lymphocyte population was gated as H1 by FSC, SSC and CD45. The T lymphocyte population was defined with CD3+ cell population in H1 gate. PD-1 subsets were stained and identified by the phenotype of CD279+ on CD4+ and CD8+ T lymphocytes. We analyzed percentages of CD4+ T lymphocytes and CD8+ T lymphocytes, and the expression of PD-1 on the membrane surface of these two cell populations. The following monoclonal antibodies were purchased from Beckman Coulter Immunology (Miami): FITC-labeled CD3 (clone No.UCHT1), PE-labeled CD4 (clone No.13B8.2), ECD-labeled CD8 (clone No.SFCI21Thy2D3), PC7-labeled CD45 (clone No.J33), APC-labeled CD279 (clone No. PD1.3).

### Statistical analysis

All statistical analysis was performed by using SPSS19.0 software (IBM, Chicago, IL, USA) and GraphPad Prism 8.0.2 (GraphPad Software Inc., La Jolla, CA). For quantitative data with a normal distribution, the t-test is used for comparison. For multiple independent samples, the One-way ANOVA test was used for comparison. Quantitative data with non-normal distribution from two independent samples was compared by a non-parametric Mann–Whitney test. For multiple samples were compared using the Kruskal & Wallis Test (non-parametric ANOVA). To evaluate correlations, Spearman’s correlation coefficient was applied. Overall survival (OS) was used and defined as the time from date of diagnosis until the date of death. The prognostic value was evaluated by Kaplan–Meier survival curves. Generate high and low PD1 expression groups based on the median survival time. Log-rank test was applied for evaluating the differences between the comparison of groups. *p* < 0.05 was considered statistically significant.

## Results

### Patient characteristics

A total of 102 patients with acute leukemia (AL) were included in this analysis, comprising 57 with Acute Myeloid Leukemia (AML) and 45 with Acute Lymphoblastic Leukemia (ALL). Additionally, 28 healthy individuals served as controls for comparison. The average age of AML patients was 52 years (range: 5–85), with 27 (47.4%) being male and 30 (52.6%) female. Among ALL patients, the average age was 26 years (range: 5–72), with 21 (46.7%) males and 24 (53.3%) females. Healthy controls had an average age of 42 years (range: 24–60), with 12 (42.9%) males and 16 (57.1%) females. Table 1 presents the clinical characteristics of the study participants. There were no significant differences in gender and age among the groups.

**Table 1:** Characteristics of healthy controls and AL patients

Group	Number	Median age (range)	Gender (M/F)	Low risk	Medium risk	High risk	No data
AL	102	43 (5–85)	48/54	5	9	36	52
AML	57	52 (5–85)	27/30	5	9	18	25
AML-ND	17	60 (16–85)	8/9	1	4	3	9
AML-CR	13	50 (5–71)	3/10	3	2	3	5
AML-NR	27	52 (17–78)	16/11	1	3	12	11
ALL	45	26 (5–72)	21/24	0	0	18	27
ALL-ND	12	33 (12–70)	6/6	0	0	6	6
ALL-CR	12	25 (7–72)	4/8	0	0	2	10
ALL-NR	21	26 (5–62)	11/10	0	0	10	11
Healthy controls	28	42 (24–60)	12/16				

*AML-ND* Newly diagnosed patients with AML, *AML-CR* Complete remission patients with AML, *AML-NR* Non-remission patients with AML, *ALL-ND* Newly diagnosed patients with ALL, *ALL-CR* Complete remission patients with ALL, *ALL-NR* Non-remission patients with ALL

### Percentage of CD4+ and CD8+ T lymphocytes in Peripheral Blood from AL Patients

**Fig. 1:**
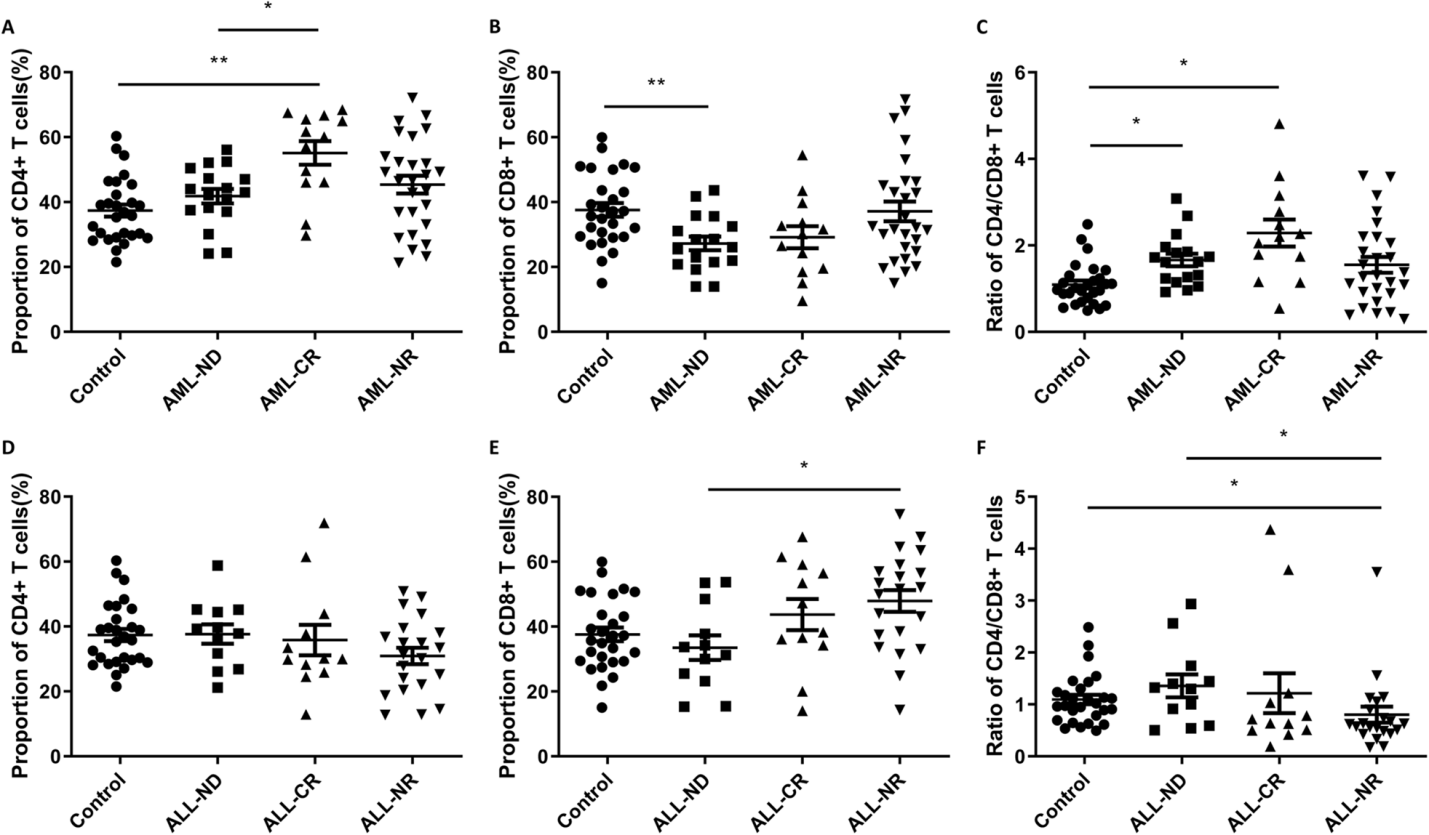
The proportion of CD4+ , CD8+ T lymphocytes to lymphocytes and the ratio of CD4/CD8 in the peripheral blood of healthy control and AL patients. **A** The proportion of CD4+ T lymphocytes to lymphocytes in the peripheral blood of healthy control and AML patients. **B** The proportion of CD8+ T lymphocytes to lymphocytes in the peripheral blood of healthy control and AML patients. **C** The ratio of CD4/CD8 in the peripheral blood of healthy control and AML patients. **D** The proportion of CD4+ T lymphocytes to lymphocytes in the peripheral blood of healthy control and ALL patients. **E** The proportion of CD8+ T lymphocytes to lymphocytes in the peripheral blood of healthy control and ALL patients. **F** The ratio of CD4/CD8 in the peripheral blood of healthy control and ALL patients. ^*^p < 0.05, ^**^p < 0.01

We observed that the proportion of CD4+ T lymphocytes in each AML group were increased in various degrees compared to the healthy control (Fig. 1A). The highest percentage of CD4+ T lymphocytes was detected in AML-CR group, and it was significantly higher (*p* = 0.0019) than healthy control (Fig. 1A). In contrast, the percentage of CD8+ T lymphocytes in AML-ND was significantly reduced (*p* = 0.0066) compared to control (Fig. 1B). We also evaluated the ratio of CD4/CD8 in peripheral blood from AL patients and healthy control, and it was significantly higher in AML-ND (*p* = 0.0124) and AML-CR (*p* = 0.0124) groups than control (Fig. 1C). We also evaluated the percentage of CD4+ T lymphocytes in ALL patients and healthy control, but the results were not statistically significant (Fig. 1D). The percentage of CD8 + T lymphocytes in AML-NR was significantly higher (*p* = 0.0390) than AML-ND (Fig. 1E). The ratio of CD4/CD8 was significantly reduced in AML-NR group compared to control (*p* = 0.0345) and AML-ND group (*p* = 0.0347) (Fig. 1F). The proportion of CD4+ and CD8+ T lymphocytes and ratio of CD4/CD8 are important indicators for evaluating immune function. Generally, the normal range of CD4/CD8 ratio is about 1.4–2.0. As shown in Fig. 1, abnormal CD4/CD8 ratio was associated with immune dysfunction in patients.

### Expression of PD-1 on CD4+ and CD8+ T lymphocytes in peripheral blood of AL patients

**Fig. 2:**
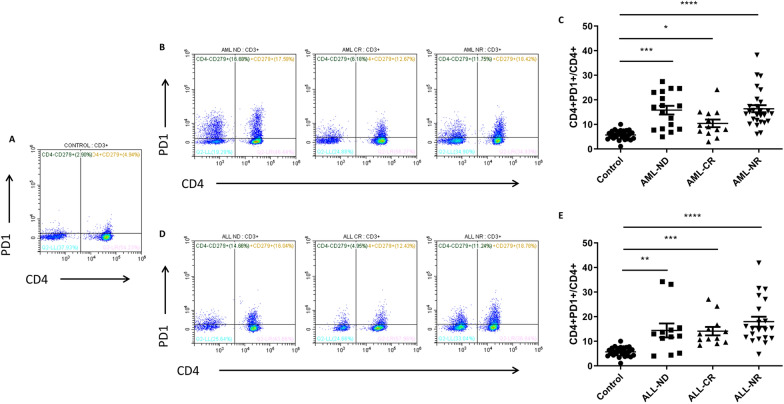
The expression of PD-1 in CD4+ T lymphocytes from AL patients and controls. **A** Representative flow cytometry plots of CD3+ CD4+ CD279+ T lymphocytes in healthy controls. **B** Representative flow cytometry plots of CD3+ CD4+ CD279+ T lymphocytes in AML patients. **C** The expression of PD-1 in CD4+ T lymphocytes from AML patients. **D** Representative flow cytometry plots of CD3+ CD4+ CD279+ T lymphocytes in ALL patients. **E** The expression of PD-1 in CD4+ T lymphocytes from ALL patients. ^****^p < 0.0001, ^***^p < 0.001, ^**^p < 0.01, ^*^p < 0.05

Our results showed that PD1 expression on CD4+ T lymphocytes was significantly increased both in AML patients and ALL patients compared to healthy controls (Fig. 2). The percentage of CD4+ PD1+ T lymphocytes in the peripheral blood of AML-CR group was significantly increased compared to controls (*p* = 0.0463) (Fig. 2A, B and C). Especially, PD-1 expression on CD4+ T lymphocytes in AML-ND (*p* < 0.0001) and AML-NR (*p* < 0.0001) groups was also significantly elevated than the control group (Fig. 2A, B and C). Similarly, we observed significant elevated percentages of CD4+ PD1+ lymphocytes in ALL-ND (*p* = 0.0023), ALL-CR (*p* = 0.0003) and ALL-NR (*p* < 0.0001) groups compared to those in the healthy control (Fig. 2A, D and E).

**Fig. 3:**
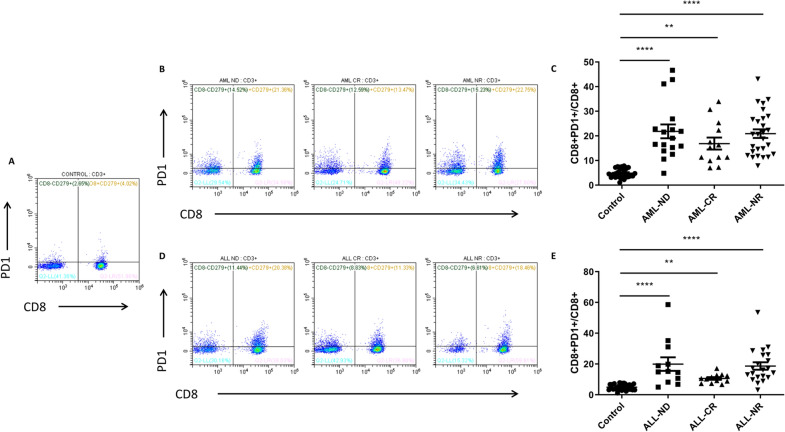
The expression of PD-1 in CD8+ T lymphocytes from AL patients and controls. **A** Representative flow cytometry plots of CD3+ CD8+ CD279+ T lymphocytes in healthy controls. **B** Representative flow cytometry plots of CD3+ CD8+ CD279+ T lymphocytes in AML patients. **C** The expression of PD-1 in CD8+ T lymphocytes from AML patients. **D** Representative flow cytometry plots of CD3+ CD8+ CD279+ T lymphocytes in ALL patients. **E** The expression of PD-1 in CD8+ T lymphocytes from ALL patients. ^****^p < 0.0001, ^**^p < 0.01

Moreover, we observed that PD1 expression on CD8+ T lymphocytes was significantly increased both in AML patients and ALL patients compared to healthy controls (Fig. 3). Levels of CD8+ PD1+ T lymphocytes in AML-ND (*p* < 0.0001), AML-CR (*p* = 0.0016) and AML-NR (*p* < 0.0001) were obviously elevated than those in controls (Fig. 3A, B and C). Percentage of CD8+ PD1+ lymphocytes in ALL-ND (*p* < 0.0001), ALL-CR group (*p* = 0.0055) and ALL-NR group (*p* < 0.0001) were significantly higher than those in controls (Fig. 3A, D and E).

### *Changes in PD-1 expression on CD4*+ *and CD8*+ *T lymphocytes of newly diagnosed AL patients after effective treatment*

**Fig. 4:**
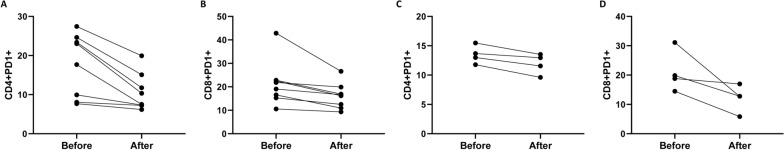
Comparison of PD-1 expression in AL patients at the time of initial diagnosis and after achieving complete remission through effective therapy. **A** Changes in CD4+ PD1+ T lymphocytes in AML patients, n = 8, *p* = 0.0040. **B** Changes in CD8+ PD1+ T lymphocytes in AML patients, n = 8, *p* = 0.0173. **C** Changes in CD4+ PD1+ T lymphocytes in ALL patients, n = 4, *p* = 0.0165. **D** Changes in CD4+ PD1+ T lymphocytes in ALL patients, n = 4, *p* = 0.0806

To further elucidate the changes in PD-1 levels on T cells in newly diagnosed AL patients after effective treatment, we assessed PD-1 expression on CD4+ and CD8+ T lymphocytes in 8 AML patients and 4 ALL patients who achieved complete remission after therapy. Our results revealed a significant decrease in PD-1 expression on both CD4+ and CD8+ T lymphocytes in AML patients after effective treatment (*p* = 0.0040 and *p* = 0.0173, Fig. 4A, B). In ALL patients, the proportion of CD4+ PD1+ T lymphocytes significantly decreased (*p* = 0.0165, Fig. 4C), while CD8+ PD1+ showed a declining trend after treatment, albeit without statistical significance (*p* = 0.0806, Fig. 4D).

### *Relationship between clinical characteristics of AL patients and PD-1 expression on CD4*+ *and CD8* + *T lymphocytes*

**Fig. 5:**
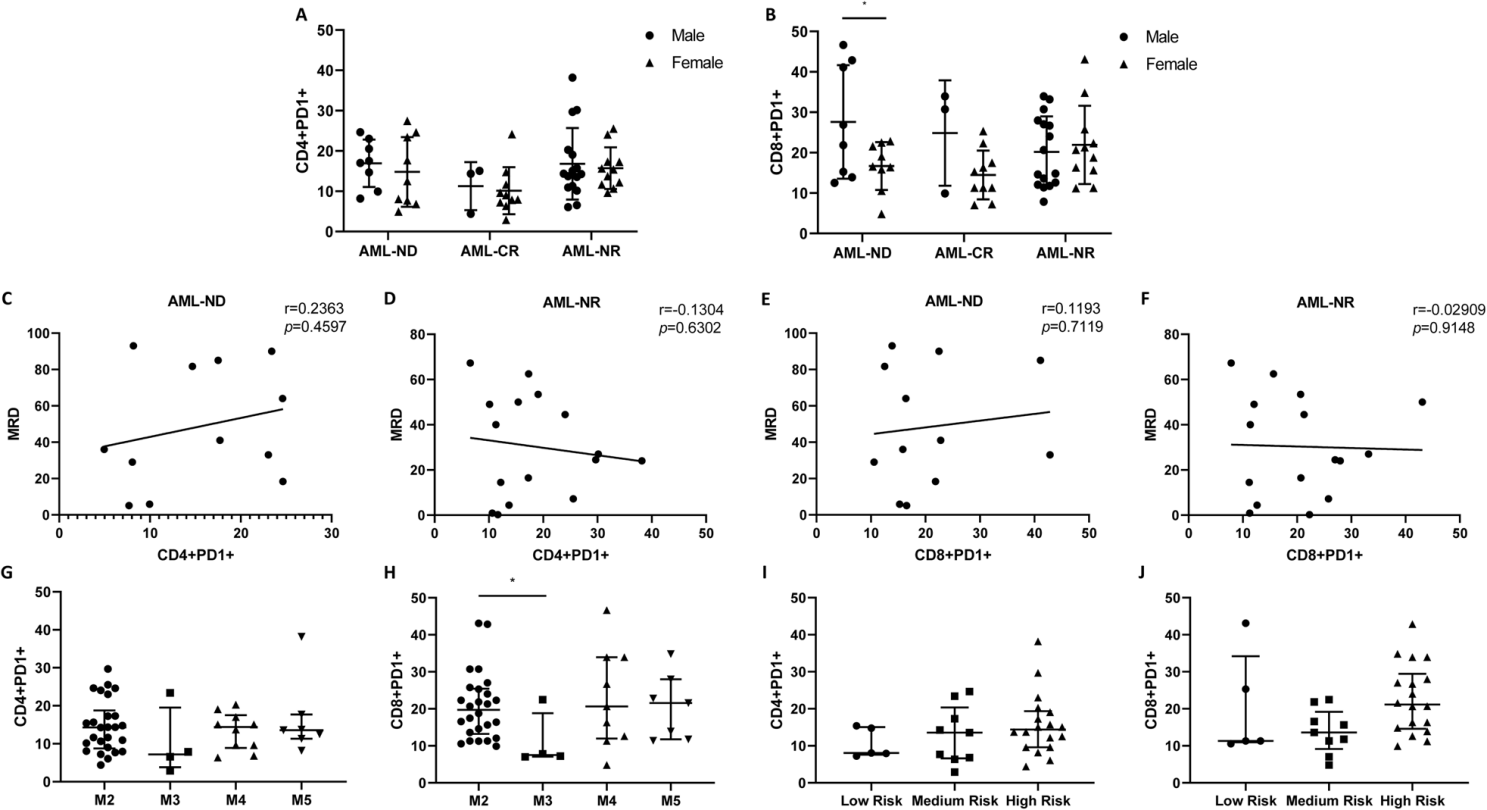
Clinical correlation of PD1 in AML patients. **A** The level of CD4+ PD1+ T lymphocytes in male and female AML patients. **B** The level of CD8+ PD1+ T lymphocytes in male and female AML patients. **C** Correlations between MRD and CD4+ PD1+ T lymphocytes in newly diagnosed patients with AML. **D** Correlations between MRD and CD4+ PD1+ T lymphocytes in non-remission patients with AML. **E** Correlations between MRD and CD8+ PD1+ T lymphocytes in newly diagnosed patients with AML. **F** Correlations between MRD and CD8+ PD1+ T lymphocytes in non-remission patients with AML. **G** The level of CD4+ PD1+ T lymphocytes in different types of AML patients. **H** The level of CD8+ PD1+ T lymphocytes in different types of AML patients. **I** The level of CD4+ PD1+ T lymphocytes in AML patients with different risk stratifications. **J** The level of CD8+ PD1+ T lymphocytes in AML patients with different risk stratifications. Each point represents an individual. The horizontal bar represents the average. ^*^p < 0.05

In this study, we grouped all patients with AL according to different types of clinical factors, including gender, minimal residual disease (MRD), disease classification, and risk stratification. The results suggested that there was no significant difference in the level of CD4+ PD1+ in the peripheral blood of male and female AML patients (Fig. 5A), but the level of CD8+ PD1 + in male patients was significantly higher than that in female patients (Fig. 5B). In addition, the correlation between PD1 and MRD was also analyzed. We found that the percentage of CD4+ PD1+ T lymphocytes in AML patients was not significantly correlated with MRD, and there was no significant correlation between CD8+ PD1+ and MRD (Fig. 5C, D, E and F). Although there was no significant difference in CD4 + PD1 + levels among AML patients of different disease types (Fig. 5G), the CD8+ PD1+ levels of M2 AML patients were significantly higher than those of M4 (*p* < 0.01) (Fig. 5H). We also analyzed the relationship between PD-1 expression and risk stratification in AML patients, which indicated that there were no significant differences in PD-1 expression on CD4+ and CD8+ T lymphocytes among low-risk, intermediate-risk, and high-risk groups (Fig. 5I and J). Additionally, we have included the comparative results of baseline characteristics, including risk stratification, for the high PD-1 and low PD-1 groups of patients, which showed that there were no significant differences in baseline characteristics, including risk stratification, between patients in the high PD-1 group and those in the low PD-1 group (Table 2 and Table 3).

**Table 2:** Comparison of baseline characteristics in low and high CD4+ PD1+ AML patients

Variable	Total number (n = 57)	Low CD4+ PD-1+ (n = 28)	High CD4+ PD-1+ (n = 29)	Statistic	p
Gender, n (%)				χ^2^ = 2.998	0.088
Male	27 (47.37)	11 (36.67)	16 (59.26)		
Female	30 (52.63)	19 (63.33)	11 (40.74)		
Risk, n (%)				–	1.000
Low risk	5 (15.62)	3 (17.65)	2 (13.33)		
Medium risk	9 (28.12)	5 (29.41)	4 (26.67)		
High risk	18 (56.25)	9 (52.94)	9 (60.00)

**Table 3:** Comparison of baseline characteristics in low and high CD8+ PD1+ AML patients

Variable	Total number (n = 57)	Low CD8+ PD-1+ (n = 28)	High CD8+ PD-1+ (n = 29)	Statistic	p
Gender, n (%)				χ^2^ = 0.449	0.503
Male	27 (47.37)	12 (42.86)	15 (51.72)		
Female	30 (52.63)	16 (57.14)	14 (48.28)		
Risk, n (%)				–	0.138
Low risk	5 (15.62)	3 (17.65)	2 (13.33)		
Medium risk	9 (28.12)	7 (41.18)	2 (13.33)		
High risk	18 (56.25)	7 (41.18)	11 (73.33)		

**Fig. 6:**
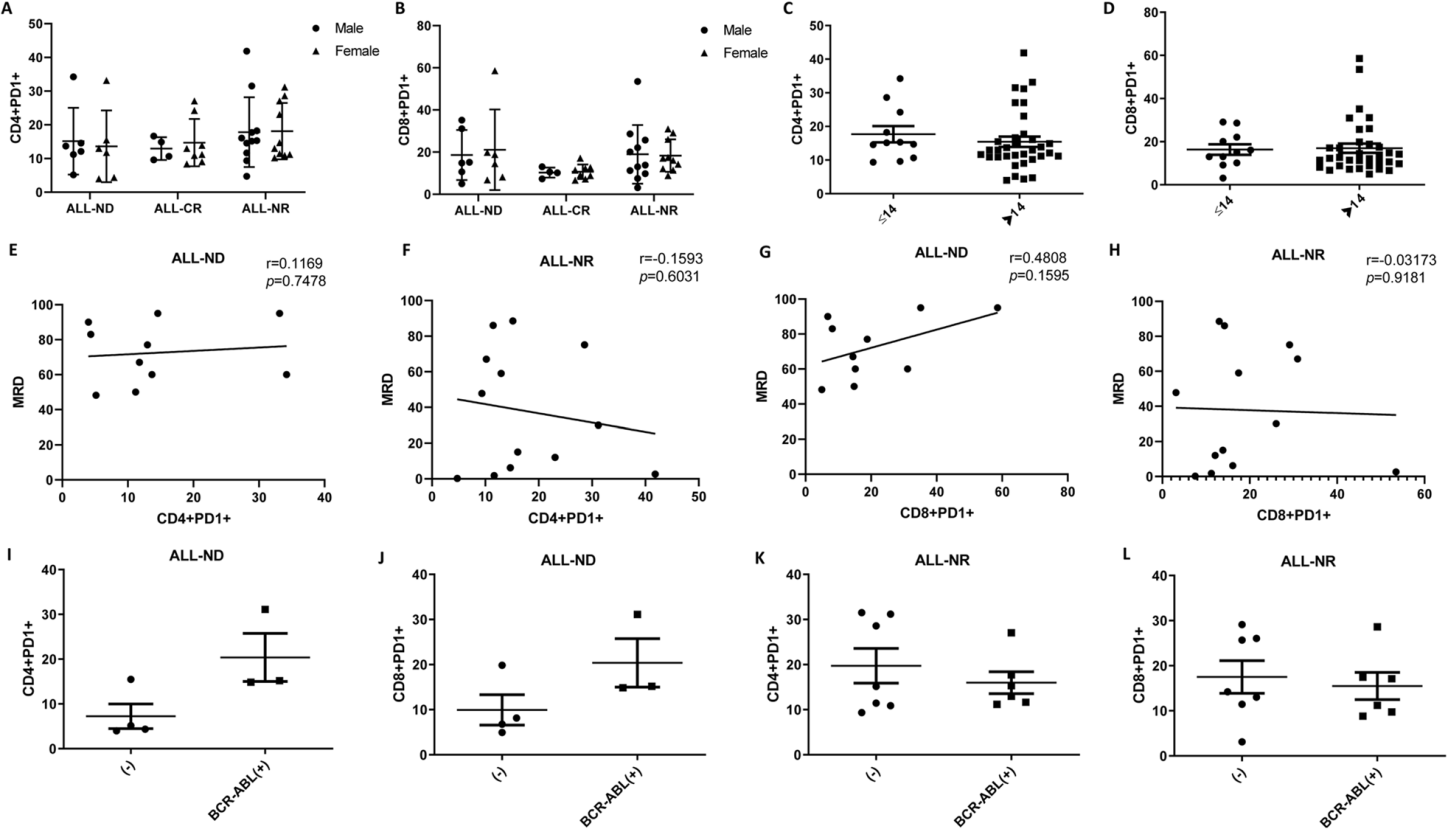
Clinical correlation of PD1 in ALL patients. **A** The level of CD4+ PD1+ T lymphocytes in male and female ALL patients. **B** The level of CD8+ PD1+ T lymphocytes in male and female ALL patients. **C** The level of CD4+ PD1+ T lymphocytes in different age groups of ALL patients. **D** The level of CD8+ PD1+ T lymphocytes in different age groups of ALL patients. **E** Correlations between MRD and CD4+ PD1+ T lymphocytes in newly diagnosed patients with ALL. **F** Correlations between MRD and CD4+ PD1+ T lymphocytes in non-remission patients with ALL. **G** Correlations between MRD and CD8+ PD1+ T lymphocytes in newly diagnosed patients with ALL. **H** Correlations between MRD and CD8+ PD1+ T lymphocytes in non-remission patients with ALL. **I** The level of CD4+ PD1+ T lymphocytes in BCR-ABL+ group of ALL-ND patients. **J** The level of CD8+ PD1+ T lymphocytes in BCR-ABL+ group of ALL-ND patients. **K** The level of CD4+ PD1+ T lymphocytes in BCR-ABL+ group of ALL-NR patients. **L** The level of CD8+ PD1+ T lymphocytes in BCR-ABL+ group of ALL-NR patients. The horizontal bar represents the average

The results showed that there was no significant difference in the levels of CD4+ PD1+ and CD8+ PD1+ T lymphocytes between male and female ALL patients (Fig. 6A, B). In addition, there was no significant difference in PD1 expression on CD4+ and CD8+ T lymphocytes neither ALL patients younger than 14 years old nor ALL patients older than 14 years old (Fig. 6C, D). Next, correlations between MRD and PD1 expression in ALL patients was analyzed. There was no relationship between the percentage of CD4 + PD-1 + T lymphocytes and MRD in peripheral blood of ALL patients (Fig. 6E, F). There was no relationship between the percentages of CD8+ PD-1+ T lymphocytes and MRD (Fig. 6G, H). Finally, we analyzed the expression of PD1 in BCR-ABL positive ALL patients. It is worth noting that we found that the levels of CD4+ PD1+ and CD8+ PD1+ in newly diagnosed ALL patients with BCR-ABL positive were higher than those in the negative group, but there was no significant difference. The opposite is true in relapsed ALL patients (Fig. 6I–L).

### Relationship between PD1 expression in peripheral circulation and survival in AL patients

**Fig. 7:**
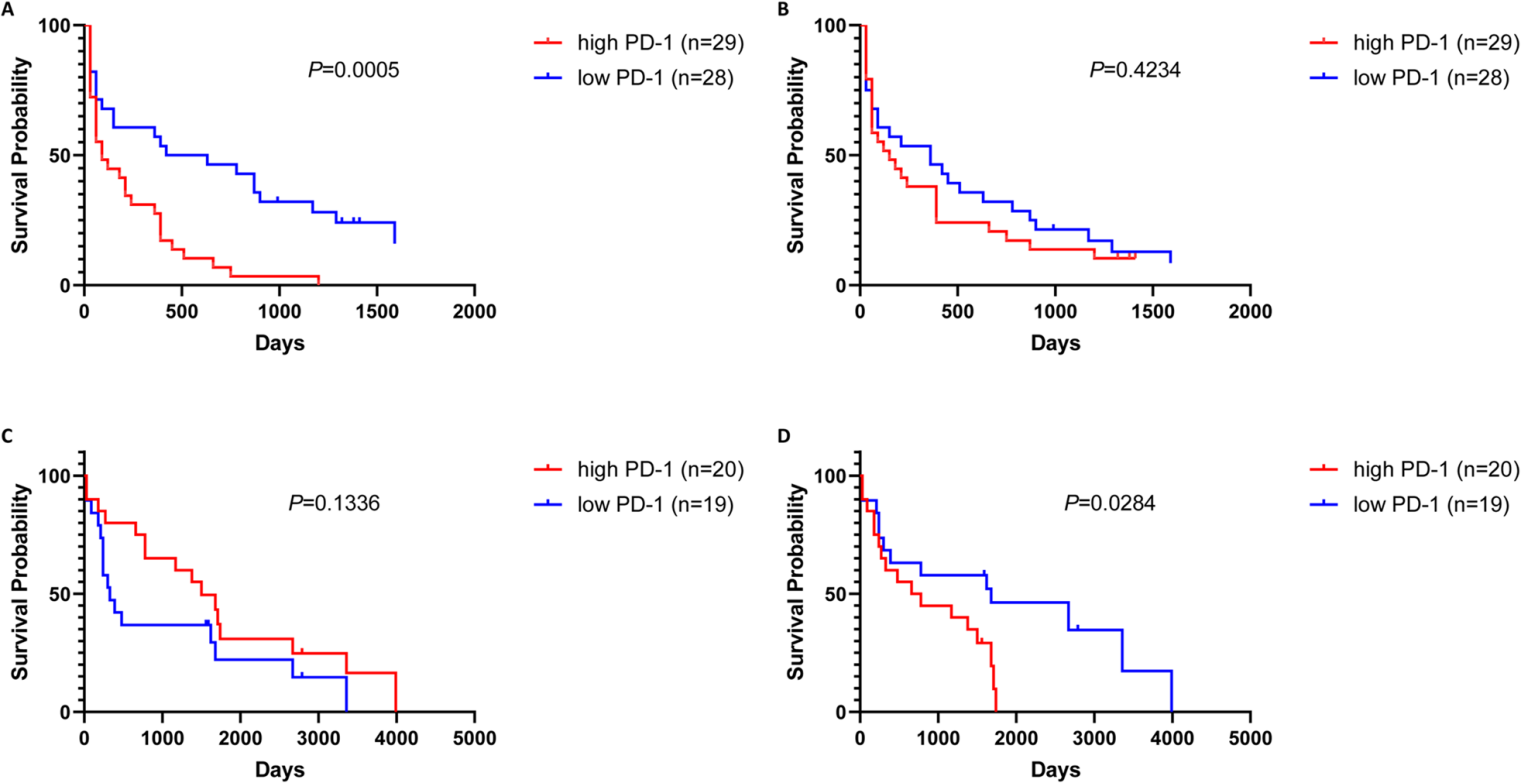
Kaplan–Meier survival curve of overall survival (OS) according to immune cell specific PD-1 expression. **A** Kaplan–Meier analysis of OS according to PD-1 expression in CD4+ T lymphocytes of AML patients. **B** Kaplan–Meier analysis of OS according to PD-1 expression in CD8+ T lymphocytes of AML patients. **C** Kaplan–Meier analysis of OS according to PD-1 expression in CD4+ T lymphocytes of ALL patients. **D** Kaplan–Meier analysis of OS according to PD-1 expression in CD8+ T lymphocytes of ALL patients

We evaluated the relationship between PD1 expression in peripheral circulation and survival in AL patients. We found that the AML patients with the higher percentage of CD4+ PD1+ in peripheral blood had shorter OS than those with the lower percentage (median 90 *vs* 525 days, *p* = 0.0005) (Fig. 7A). Additionally, there is no significant difference in OS of AML patients with high levels of CD8+ PD1+ compared with low levels (median 150 *vs* 360 days, *p* = 0.4234) (Fig. 7B). On the other hand, the median OS of patients with ALL with a high level of CD4+ PD1+ T lymphocytes in the peripheral blood was 1440 days and the low level was 330 days, but the difference between the two was not statistically significant (*p* = 0.1336) (Fig. 7C). ALL patients with low levels of CD8+ PD1+ in the peripheral blood have a significant survival advantage over patients with high levels of CD8+ PD1+ in the peripheral circulation (median 720 *vs* 1620 days, *p* = 0.0284) (Fig. 7D). To determine whether aberrant PD-1 expression is an independent factor affecting prognosis in AL patients. Univariate analysis was used, and the results were shown in Table 4. Higher CD4+ PD1+ (*p* = 0.042) in AML patients and higher CD8+ PD1+ (*p* = 0.019) in ALL patients were found to predict OS (Table 4). Together, these results indicate that the high level of CD4+ PD1+ T lymphocytes and CD8+ PD1+ were respectively poor prognostic indicators of AML patients and ALL patients.

**Table 4:** Cox regression analysis for OS

Groups	Variable	HR	95% CI	p value
AML	CD4+ PD1+	1.791	1.021–3.142	0.042
	CD8+ PD1+	1.742	0.987–3.075	0.055
ALL	CD4+ PD1+	0.775	0.381–1.577	0.483
	CD8+ PD1+	2.675	1.174–6.095	0.019

## Discussion

Traditional therapies combined with immune checkpoint inhibitors including anti-PD-1 antibody have shown better therapeutic efficacy in a variety of cancer types, including acute leukemia [16–18]. It is known that the occurrence and development of acute leukemia is closely related to the reduction of the body’s immune level, especially the abnormality of cellular immunity. T lymphocytes perform cellular immune function, and patients with leukemia are often accompanied by changes in the number of T cell subsets and functional impairment. CD3+ cells are usually used to define the total number of T lymphocytes. CD4+ cells are the main response cells in the immune response. CD8+ cells can produce cell-mediated cytotoxicity on target cells, and at the same time have a regulatory immunosuppressive effect on CD4+ cells [19–21]. In the present study, we used flow cytometry to evaluate PD-1 expression on the surface of CD4+ and CD8+ T lymphocytes in the peripheral circulation of AML and ALL patients and its clinical significance. Our results showed that the CD4/CD8 ratio in the peripheral circulation of AML patients and newly diagnosed ALL patients was increased to varying degrees compared to healthy controls. The proportion of CD4+ and CD8+ T lymphocytes and ratio of CD4/CD8 are important indicators for evaluating immune function. Generally, the normal range of CD4/CD8 ratio is about 1.4–2.0. Abnormal CD4/CD8 ratio was associated with immune dysfunction in patients, the unstable CD4+ /CD8+ ratio was not conducive to the balance of cellular immune responses.

Recent studies have found that the PD-1 expression is up-regulated in lung cancer, gastric cancer, hepatocellular carcinoma, multiple myeloma, breast cancer, renal cell carcinoma and melanoma [22–28]. The high expression of PD-1 continuously activates the PD-1/PD-L1 signaling pathway, thereby inhibiting various signaling pathways. In addition, some studies have shown that high expression of PD-1 may be a poor prognostic factor in some malignant tumors such as lymphoma [29, 30], lung cancer [31] and breast cancer [32]. At present, PD-1/PD-L1 monoclonal antibodies have made breakthroughs in clinical trials for the treatment of non-small cell lung cancer, which further indicates that PD-1 and its ligands play an important role in anti-cancer therapy. It also provides a new targeted therapy idea for the first and second-line treatment of malignant tumors.

In hematological malignancies, PD-1 expression has been reported increased in patients with Hodgkin’s lymphoma, diffuse large B-cell lymphoma and chronic lymphocytic leukemia [33–35]. Furthermore, PD-1+ T cells have also been proven in follicular lymphoma as an independent prognostic factor of overall survival [36, 37]. As far as the field of leukemia is concerned, current studies have reported that PD-1 is highly expressed in chronic lymphocytic leukemia and AML patients, and the expression level correlates with prognosis. However, clinical Implications of aberrant PD-1 expression in peripheral CD4+ and CD8+ T lymphocytes of AML and ALL patients in assessing the prognosis of diseases, remains inconclusive. The differential expression of PD-1 on different types of T cells in the peripheral circulation of AL patients and its relationship with the clinical characteristics and prognosis are still worthy of further investigation.

Our results indicated that expression levels of PD-1 on CD4+ and CD8+ T lymphocytes were significantly increased in newly diagnosed and non-remission patients compared to healthy controls both in AML and ALL patients. According to our analysis, the relationship between high PD1 expression on different T cell types and prognosis is different. To further investigate the clinical significance of PD-1 in AL, this study first analyzed the relationship between the expression of PD-1 and its clinical characteristics. Except for the increased CD8+ PD1+ levels in M2 AML patients, there was no significant association between the most common clinical indications and PD-1 expression in ALL and AML patients, indicating that PD-1 expression is not affected by general clinical manifestations and common hematologic indicators. The high level of CD4+ PD1+ and CD8+ PD1 + T lymphocytes were respectively poor prognostic indicators of AML patients and ALL patients but had no significant correlation with most common clinical risks. We found that the AML patients with the higher percentage of CD4+ PD1+ in peripheral blood had shorter OS than those with the lower percentage. ALL patients with low levels of CD8+ PD1+ in the peripheral blood have a significant survival advantage over patients with high levels of CD8+ PD1+ in the peripheral circulation. Together, these results were indicated that the high level of CD4+ PD1+ T lymphocytes and CD8+ PD1+ were respectively poor prognostic indicators of AML patients and ALL patients. High expression of PD-1 in the peripheral blood of AML and ALL patients was related to their poor prognosis.

To conclude, our evidence may be limited though, but preliminarily reveals the expression level of PD-1 in peripheral circulation of newly diagnosed or non-remission AML and ALL patients was significantly higher than healthy persons. The high level of CD4+ PD1+ and CD8+ PD1+ T lymphocytes were respectively poor prognostic indicators of AML patients and ALL patients but had no significant correlation with most common clinical risks. Aberrant PD-1 expression correlates with the prognosis of AL patient and may thus serve as poor prognostic indicators. Immunotherapy using PD-1 inhibitors may be a promising strategy for AML and ALL patients with peripheral circulating CD4+ PD1+ and CD8+ PD1+ T lymphocytes positively expressed, respectively.

## Data Availability

The data used to support the findings of this study are available from the corresponding author upon request.
